# Supplementary material for: A high-resolution mRNA expression time course of embryonic development in zebrafish
Source: eLife. 2017 Nov 16;6:e30860. doi: 10.7554/eLife.30860 (PMC5690287; doi:10.7554/eLife.30860)
Supplement: Supplementary file 6. [file elife-30860-supp6.zip › biolayout-clusters-files/Cluster103-genes.html]

Cluster103


# Cluster103: Genes

| Ensembl ID | Gene Name | Chr | Start | End | Biotype |
| --- | --- | --- | --- | --- | --- |
| ENSDARG00000069828 | ABHD12B (1 of many) | 13 | 9546521 | 9554556 | protein\_coding |
| ENSDARG00000037679 | MPC2 (1 of many) | 1 | 9999732 | 10012492 | protein\_coding |
| ENSDARG00000024278 | adh8b | 13 | 12500748 | 12514277 | protein\_coding |
| ENSDARG00000035603 | dao.2 | 5 | 19667160 | 19681865 | protein\_coding |
| ENSDARG00000104474 | il6r | 16 | 18402446 | 18420775 | protein\_coding |
| ENSDARG00000045516 | itih2 | 4 | 25198714 | 25226770 | protein\_coding |
| ENSDARG00000022372 | kng1 | 22 | 36898335 | 36935388 | protein\_coding |
| ENSDARG00000054319 | oxct1b | 21 | 20734446 | 20749252 | protein\_coding |
| ENSDARG00000104207 | pcyt1bb | 21 | 40595568 | 40607603 | protein\_coding |
| ENSDARG00000090286 | serpina1 | 20 | 10556860 | 10560171 | protein\_coding |
| ENSDARG00000090850 | serpina1l | 20 | 10550785 | 10553957 | protein\_coding |
| ENSDARG00000042684 | serpinc1 | 22 | 15995941 | 16001921 | protein\_coding |
| ENSDARG00000042807 | slc16a4 | 8 | 24725902 | 24760750 | protein\_coding |
